# Neutrophils in COVID-19

**DOI:** 10.3389/fimmu.2021.652470

**Published:** 2021-03-25

**Authors:** Nico Reusch, Elena De Domenico, Lorenzo Bonaguro, Jonas Schulte-Schrepping, Kevin Baßler, Joachim L. Schultze, Anna C. Aschenbrenner

**Affiliations:** ^1^ Systems Medicine, German Center for Neurodegenerative Diseases (DZNE), Bonn, Germany; ^2^ Genomics & Immunoregulation, Life & Medical Sciences (LIMES) Institute, University of Bonn, Bonn, Germany; ^3^ German Center for Neurodegenerative Diseases (DZNE), PRECISE Platform for Genomics and Epigenomics at DZNE, University of Bonn, Bonn, Germany; ^4^ Department of Internal Medicine and Radboud Center for Infectious Diseases, Radboud University Medical Center, Nijmegen, Netherlands

**Keywords:** viral infection, SARS-CoV-2, COVID-19, granulocytes, neutrophils, scRNA-seq, clinical trials

## Abstract

Strong evidence has been accumulated since the beginning of the COVID-19 pandemic that neutrophils play an important role in the pathophysiology, particularly in those with severe disease courses. While originally considered to be a rather homogeneous cell type, recent attention to neutrophils has uncovered their fascinating transcriptional and functional diversity as well as their developmental trajectories. These new findings are important to better understand the many facets of neutrophil involvement not only in COVID-19 but also many other acute or chronic inflammatory diseases, both communicable and non-communicable. Here, we highlight the observed immune deviation of neutrophils in COVID-19 and summarize several promising therapeutic attempts to precisely target neutrophils and their reactivity in patients with COVID-19.

## Introduction

Coronavirus disease 2019 (COVID-19), the disease elicited by SARS-CoV-2 infection, has been diagnosed in over 111 million patients and led to over 2.4 million deaths during the 2020 pandemic (WHO, covid19.who.int, as of February 24th, 2021). Early on, increased neutrophil counts in the blood of severely affected individuals were noted as a major clinical feature of this novel disease (1). In combination with the concomitant lymphopenia, an elevated neutrophil-to-lymphocyte ratio has emerged as a hallmark of severe COVID-19 (2–4).

**Figure 1:**
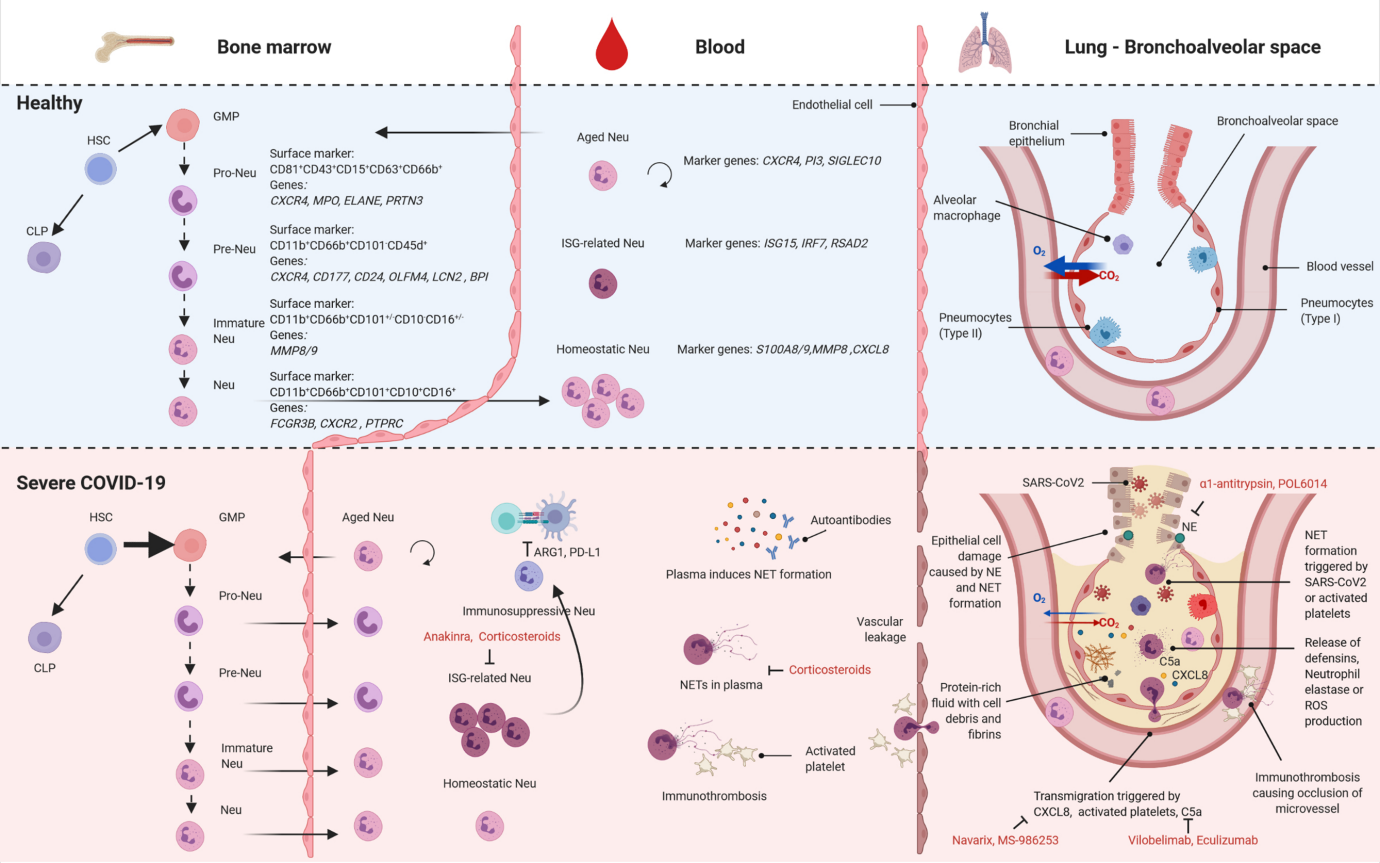
Neutrophil subsets in health and severe COVID-19. Overview of the different subsets of neutrophils found in bone marrow (left), blood (center) and lung (bronchoalveolar space, right) in health (top) and severe COVID-19 (bottom). HSC, hematopoietic stem cells; CLP, common lymphoid progenitors; GMP, granulocyte-monocyte progenitor cell; Neu, neutrophil; ISG, interferon-stimulated genes; NET, neutrophil associated extracellular trap; ROS, reactive oxygen species; created with BioRender.com.

Historically, many studies investigating circulating immune cells in disease have focused on the analysis of peripheral blood mononuclear cells excluding neutrophils and other granulocytes. Thus, knowledge on this most abundant but also technically challenging immune cell fraction in blood is lagging behind (5). Recent advances in single-cell omics technologies have opened up new possibilities to study this cell population in humans, especially in pathological contexts, challenging the understanding that neutrophils are a homogeneous population of short-lived cells (6, 7). As part of the innate immune system, granulocytes are among the first cells recruited to a site of infection and key in shaping the early response to an insult as well as in mediating between the innate and adaptive arm of the immune system (8). Yet, if not properly regulated, the powerful effector functions of these cells can lead to tissue damage (8). Here, we highlight key features and latest findings of human neutrophil biology with a special focus on COVID-19 (**Figure 1**).

## Neutrophil Ontology

Lately, multiple studies delineated the extensive heterogeneity of neutrophils. Different subsets have been identified comprising maturation stages, but also different cellular activation states (9–11). In particular, granulocyte-monocyte progenitor (GMP) cells have been described as a heterogeneous blend comprising a population of neutrophil-committed progenitors termed as pro-neutrophils (pro-Neu) (CD81^+^CD43^+^CD15^+^CD63^+^CD66b^+^), which sequentially differentiate into lineage-committed precursors (pre-Neu) (CD11b^+^CD66b^+^CD101^-^CD45d^+^), immature neutrophils (CD11b^+^CD66b^+^CD101^+/-^CD10^-^CD16^+/-^) and mature neutrophils (CD11b^+^CD66b^+^CD101^+^CD10^+^CD16^+^) (10–14).

This maturation process takes place in the bone marrow (BM) and the differentiation stages are characterized by specific transcriptional waves. Pro- and pre-Neu both present a high proliferation signature which diminishes in mature neutrophils (6). The genes that best differentiate the subsets are encoding components of their characteristic granules. Pro-Neu mostly express azurophilic granule genes (e.g. *AZU1*), followed by genes of specific granules such as *LTF* in pre-Neu and finally gelatinase and secretory granule genes in mature neutrophils (6). Interestingly, the majority of proteins found in neutrophils are generated in early differentiation stages and stored in granules. This leads to a decrease in mRNA content over their course of life (6) and thus a potential discrepancy between protein and gene expression (15). A switch in chemokine receptor expression from CXCR4 to CXCR2, finally, leads to the egress of mature neutrophils from the BM into the circulation (16, 17). This process has recently been linked to the circadian rhythm with a peak of freshly released neutrophils at night (18).

Mature neutrophils in circulation under homeostasis have been subdivided into three distinct subsets: homeostatic, which form the majority, aged and interferon-stimulated genes (ISG)-related neutrophils (6). Once in circulation, neutrophils either patrol the vasculature or migrate into different organs (7). Just as the downregulation of CXCR4 is responsible for the egress of neutrophils from the BM, the re-expression of CXCR4 on aged neutrophils will eventually lead to the migration back into the BM, spleen, or liver where aged neutrophils are phagocytosed by macrophages (18, 19). Previously extravasated neutrophils may also re-enter circulation, migrate to the pulmonary microcirculation where they upregulate CXCR4 to subsequently enter the BM for clearance (20). Further, the lung microvasculature has been recognized as a functional immune niche (21, 22). Of note, most studies investigating the differentiation processes have been performed in mouse models and might thus not be fully translatable to human neutrophils (23). However, hematopoietic structures including progenitor populations in fetal bone marrow and umbilical blood (10) are highly similar between mouse and human (24).

## Neutrophil Function in Health & Disease

Neutrophils play a crucial role in the first line of cell-mediated defense against microbes. They phagocytose bacteria and clear them by fusion with their cytoplasmic granules containing proteases, defensins, antimicrobial peptides or reactive oxygen species (ROS). Additionally, they can form neutrophil extracellular traps (NETs), in which parts of the nucleus together with granules are actively released (25). These mechanisms are differentially active in the above mentioned neutrophil subsets. Phagocytosis capacity as well as ROS production increase with maturation stage (6, 12). Aging of neutrophils during circulation leads to a gradual degranulation that decreases their capacity for NET formation (15).

Neutrophils perform their effector activities after migration into peripheral tissues. Circulating CXCR1^+^CXCR2^+^ mature neutrophils are orchestrated to tissues by gradients of CXCL1, CXCL2, CXCL8, CCL3, and CCL2 (26). Binding of these chemokines does not only guide the localization, but also activates them as demonstrated by CXCL8 that can elicit ROS production and induce L-Selectin shedding (27). Other important inflammatory mediators such as the complement component C5a contribute to the recruitment of neutrophils to sites of infection (28) and to the activation of NET formation when primed by interferons (29). Additionally, a growing body of evidence shows a link between neutrophils and activated platelets that guide their migration into inflamed tissue and induce NET formation (30, 31). Together, neutrophils and activated platelets can elicit a process termed immunothrombosis in blood vessels where they contribute to the formation of a fibrin mesh that can trap pathogens (32), but more importantly intravascular NET formation by itself can lead to the fibrin-independent occlusion of microvessels causing massive cell death in affected areas (33).

At the location of infection, neutrophils can exert their antimicrobial activities. Here, the HIF-NFκB axis represents an additional checkpoint to prevent unwanted effector functions. HIF-1α is expressed at low levels in circulating neutrophils, but upregulated in hypoxic conditions of inflamed tissue (34). The HIF pathway has been described to increase the expression of antimicrobial peptides and promote degranulation, but on the other hand might inhibit ROS production and NET formation (35). Moreover, HIF signaling inhibits apoptosis of neutrophils prolonging their lifetime in inflamed tissue (36).

The neutrophil defense mechanisms can come at the high price of collateral damage. Apart from the common utilization of their granules and produced ROS to clear pathogens upon phagocytosis, these effector molecules can be released and target the surrounding tissue (37). In acute respiratory distress syndrome for instance, excessive neutrophil activation can lead to an increased permeability of blood vessels due to released defensins and neutrophil elastase (NE) (38, 39). In addition, NET formation may not only activate alveolar macrophages for clearance (40), but also largely contributes to the activation of endothelial cells exacerbating the inflammatory circuit (37) or can actively damage endothelial tissue (41).

The functional capacity and extravasation behaviour of neutrophils is tightly coupled to a cell-intrinsic circadian process (15, 18). This homeostatic program ensures protection from excessive inflammation and vascular damage by the gradual loss of granule proteins that reduces the neutrophils’ toxic activities (15). Further, aged neutrophils exhibit declined ability to enter inflammatory sites and favor homeostatic clearance into non-inflamed tissues (18, 23). Neutrophils may not only damage host tissue, but they can also suppress the fine-tuned adaptive immune response. So-called granulocytic myeloid-derived suppressor cells (MDSC), originally identified in cancer (42, 43), have now additionally been described in multiple viral chronic infections, such as HCV (44) and HIV (45–47), to inhibit lymphocyte proliferation *via* depletion of arginine by Arginase-1 (48) or through the expression of PD-L1 (46). Neutrophils with suppressive features have further been found in several mostly chronic non-communicable diseases, including systemic lupus erythematosus and rheumatoid arthritis patients. This type of neutrophil co‐segregates with mononuclear cells after density gradient centrifugation of blood, thus termed low-density neutrophils (LDNs) (49). Interestingly, also LDNs with a pro-inflammatory phenotype were reported in systemic autoimmune diseases and are characterized by enhanced levels of cytokine secretion (e.g. type I interferon) and NET formation (23, 50). The distinction between immunosuppressive and pro-inflammatory LDNs is still solely based on functional assays since no cellular markers were identified to distinguish the two subsets, underlining the need for further studies to fully understand their origin and function (23).

Besides activating the circulating pool of mature neutrophils, severe insults such as sepsis, trauma and viral infections can induce emergency granulopoiesis, a hematopoietic response program that rapidly increases the *de novo* production of neutrophils to cope with increasing demands. This mechanism results in the presence of both immature neutrophils and mature populations in the peripheral blood, that can act either immunosuppressive or pro-inflammatory (11, 51). Despite the limited knowledge about the respective contribution of mature and immature neutrophils to the immune response and their distinct features, the clinical interest in these cells is growing due to their increasingly apparent correlation with disease severity and/or response to treatment in many pathologies, such as sepsis and severe influenza (11, 52–54). The phenomenon of emergency granulopoiesis and the availability of many freshly generated neutrophils may increase their destructive capacity.

## Neutrophils in COVID-19

Pathophysiology of severe COVID-19 is marked by altered neutrophil abundance, phenotype and functionality. Upon SARS-CoV-2 infection, elevated numbers of neutrophils have been observed in the nasopharyngeal epithelium (55) and later in the more distal parts of the lung (56). Increased neutrophil counts have also been detected in the clinics as a feature of COVID-19 in the blood (2–4) and neutrophil activation signatures are a prominent feature of blood transcriptomes of severe cases (57, 58). Further analysis of granulocyte samples attributed this alteration to gene expression changes in these cells, opposed to a mere change in abundance in circulation, with prominent features of pre-/immature neutrophil markers upregulated in severe vs. mild COVID-19 patients (58). In addition, plasma levels of RETN, HGF, and LCN2, typically produced by neutrophils, were recently proposed as predictive for critical illness and mortality (57).

Single-cell RNA sequencing (scRNA-seq) analysis of whole blood samples after erythrocyte lysis allowed a comprehensive investigation of the transcriptional regulation of the neutrophil compartment in the blood of COVID-19 patients (13). Subclustering and marker-based cell annotation revealed extensive heterogeneity with transcriptionally distinct pro- and pre-Neu and seven mature neutrophil clusters. The mature clusters show a strong shift towards more ISG-related neutrophils correlating with disease severity. The appearance of pro- and pre-Neu specifically in the circulation of severe COVID-19 patients at later stages of the disease is a clear indicator of emergency myelopoiesis (13, 59). The pro-Neu are characterized by genes involved in NET formation including *MPO*, *ELANE*, *PRTN3* whereas the pre-Neu present with *CD177*, *CD24*, *OLFM4*, *LCN2* and *BPI* expression, genes which have been associated with poor outcome in sepsis (60, 61). Interestingly, these markers can already be found by whole blood bulk RNA-sequencing differentiating severe from mild patients (58). Moreover, two mature neutrophil clusters were found to be specific for severe disease course and resemble phenotypes of granulocytic MDSCs (62) and PD-L1^+^ neutrophils after LPS challenge *in vivo* (63). Later studies were able to isolate MDSC-like neutrophils from COVID-19 patients and provided evidence for their capacity to inhibit T cell proliferation and IFNγ production (64, 65). As lower levels of IFNγ production by lymphocytes have been reported early on (66), these immunosuppressive neutrophils may contribute to this phenotype.

Unsupervised clustering analysis of mass cytometry data of whole blood samples of COVID-19 patients confirmed the heterogeneity among neutrophils observed in single-cell transcriptomics data and corroborated disease-specific alterations in COVID-19 on protein level (13). Severe COVID-19 is associated with a striking increase in immature neutrophil populations defined by their expression of CD11b, CD16, CD24, CD34 and CD38 and showing features of recent activation, such as amplified surface expression of CD64, RANK and RANKL and reduced CD62L expression. Spectral flow cytometry furthermore confirmed the substantial increase in immature/pre-Neu, here defined by CD10^lo^CD101^-^ expression, in patients with severe COVID-19 (59). In addition, elevated PD-L1 surface expression and CD62L downregulation in neutrophils supports the transcriptional signature of a suppressive phenotype of neutrophils in severe COVID-19 (13).

scRNA-seq analysis of the low density cell fraction derived from blood samples of COVID-19 patients by density gradient centrifugation revealed the presence of LDN typical for chronic infectious diseases also in the circulation in severe cases of COVID-19 (13). Another study reported lower granularity of neutrophils from COVID-19 patients that might explain the lower density (67). Detailed analysis of LDN partially reflected the transcriptional heterogeneity observed in whole blood neutrophil samples with distinct pro-, pre- and mature neutrophil clusters. COVID-19-relevant expression patterns such as expression of genes involved in NET formation as well as of ISG (*ISG15*, *IFITM1/3*, and *RSAD2*) were identified in LDN. While all LDN expressed high levels of *S100A8* and *S100A9*, alarmins described to have predictive functions for COVID-19 severity (59, 68), *CD274* (PD-L1*)* and *ARG*1 were also expressed in distinct LDN subsets. These findings showed that LDNs are not transcriptionally uniform, but rather present different subsets of neutrophils that are similar in their density profiles.

Functional analysis did not show an alteration of the phagocytosis capacity of neutrophils from peripheral blood from mild and severe COVID-19 patients (13). However, ROS production upon co-cultivation with *Escherichia coli* or stimulation with Phorbol-12-myristat-13-acetate was significantly reduced in neutrophils from severe as compared to mild COVID-19 patients or controls (13). Interestingly, the degranulation of primary granules as depicted by increased levels of cell surface CD63 was shown to be elevated in severe COVID-19 patients which is probably the cause for their increased serum levels of MPO and NE (69). Markers for the gelatinase granules (CD11b) and secondary granules (CD66b) did not change on the surface of neutrophils from healthy and diseased patients (69).

A heightened capacity for NET formation was also functionally validated. Not only were increased levels of NETs reported in plasma of COVID-19 patients, which positively correlated with disease severity, but autopsies revealed clear enrichment of NETs in patients’ lungs (67, 70). The increased capacity for NET formation in COVID-19 can be due to inherently different neutrophil subsets (13) or by inflammatory mediators in the circulation. Indeed, serum from severe COVID-19 patients induces NET formation (67, 71), which might at least in part be explained by autoantibodies as the incubation of the IgG antibody fraction from severe COVID-19 patients was sufficient to induce NET formation in neutrophils from healthy patients (72). At least 50% of the patients in this study were positive for anti-phospholipid autoantibodies that may explain this effect (72). NET formation in the lungs may additionally be triggered by direct contact of neutrophils with SARS-CoV-2 as *in vitro* stimulations with the virus induced NET formation likely *via* TLR7 signalling (70, 71, 73). NETs were also suggested to kill lung epithelial cells (70). Another pathophysiological mechanism linked to neutrophils in severe COVID-19 is the increase in platelet-neutrophil aggregates that contribute to increased levels of NET formation (67, 74, 75). Immunothrombosis can lead to an occlusion of microvessels in the lung causing cell death in affected areas contributing to worsened respiratory function of the organ. The risk of immunothrombosis is further increased by vasoconstriction caused by the cytokine release syndrome observed in severe COVID-19 (76) or by hypoxic pulmonary vasoconstriction which is possibly dysregulated in severe COVID-19 (77, 78). As mentioned above, hypoxia can contribute to the activation of neutrophils further promoting the vicious cycle of neutrophil reactivity in damaged lung tissue. Moreover, the decreased oxygen saturation in blood of severe COVID-19 patients might activate HIF-1a signaling in circulation and contribute to excessive neutrophil function in COVID-19 patients. As proposed by others, the role of HIF-1a in COVID-19 needs further attention (79, 80). Extensive immunothrombosis can also be the cause of severe cardiovascular events seen in severe COVID-19 (67, 81–86). Since NET formation was not yet functionally linked to the different subsets of neutrophils that were described by scRNA-seq it will be interesting to study which role each subset plays.

Further scRNA-seq studies have identified an increase in tissue-infiltrating neutrophils in COVID-19 in the upper airways (55) and the bronchoalveolar space (56, 87) that correlate with disease severity. The neutrophils in the bronchoalveolar space present as a heterogeneous population with very few pre-/pro-Neu and three mature subsets. These were differentiated into *S100A12* ^hi^ neutrophils (specifically up in critical COVID-19), *CXCL8* ^hi^ neutrophils and *CD74* ^hi^ neutrophils. In contrast, a study based on flow cytometry data did not find increased neutrophil counts in severe COVID-19 patients in bronchoalveolar lavage as compared to other ICU patients (either non-pneumonia or pneumonia) (88). However, a separation between early and late sampling reveals a strong increase in neutrophils at later time points of COVID-19 in the lung indicating a possible bias due to sampling time.

## Treatment of Neutrophil-Associated Damage in COVID-19

In light of the key role of neutrophil-induced tissue damage in the pathology of COVID-19, targeting the effector functions or the extravasation of neutrophils in the lungs constitutes a promising opportunity for pharmacological intervention (89). Beyond the current SARS-CoV-2 pandemic, a number of ongoing pharmacological studies aim to manipulate neutrophil activity in several pulmonary diseases characterized by an excessive neutrophil-mediated tissue damage (e.g. COPD and bronchiectasis) (90–92). These studies mostly target the process of neutrophil recruitment and chemotaxis by inhibiting the chemokine receptor CXCR2. An example is Navarixin (MK-7123/SCH 527123) which showed the potential to improve lung function in COPD patients (90).

Due to the current need of additional therapeutic options for severe COVID-19 patients, several strategies are under clinical investigation targeting different aspects of the host response to SARS-CoV-2 infection (89). NE is an enzyme which contributes to SARS-CoV-2 infectivity by proteolytic priming of the viral glycoproteins enabling membrane fusion in the host and mediating the damage to the infected lungs (93). Furthermore, NE activity was found to be increased in the plasma of COVID-19 patients having a pro-inflammatory and prothrombotic effect, exemplified by the occlusion of the pulmonary microvasculature (75, 94). Thus, targeting this enzyme presents a valuable option as therapeutic intervention, especially by pulmonary administration of the developed NE inhibitors, helping to achieve a broader therapeutic window by avoiding excessive side effects. The CXCL8/CXCR2 axis, involved in neutrophil migration, is also under exploration and a CXCL8 blocking antibody (MS-986253) is currently tested in clinical trials in COVID-19 patients. Further, the aforementioned Navarixin, together with other CXCR2 inhibitors, has been proposed as treatment for COVID-19 (95). Another opportunity to block neutrophil hyperactivation is acting on IL-1β/IL-1R interaction; anakinra (IL-1R inhibitor) is currently under clinical investigation showing promising preliminary results (96–99), whereas canakinumab (IL-1β inhibitor) failed to ameliorate the outcome of severe COVID-19 patients (https://www.novartis.com/news/media-releases/novartis-provides-update-can-covid-trial-hospitalized-patients-covid-19-pneumonia-and-cytokine-release-syndrome-crs). The complement C5a-C5aR1 interaction also appears to be a candidate in the modulation of neutrophil-induced tissue damage (100). The C5a molecule was found to increase proportionally with COVID-19 severity (101) and is known to induce neutrophil recruitment as well as activation by binding the C5aR1 receptor (28). Preliminary clinical studies for IFX-1 (vilobelimab), a monoclonal antibody against C5a, and Solris (eculizumab), a C5 blocking antibody, showed promising results in the management of severe COVID-19 (102, 103).

Corticosteroids are a currently recommended treatment for severe COVID-19 (104) shown to reduce neutrophils’ respiratory burst and recruitment to the inflamed tissue at system level (105) and inhaled glucocorticoids have been shown to reduce NET formation in asthma patients (106). Additionally, they might have a pro-inflammatory and anti-apoptotic effect on neutrophils by reducing the levels of both, the secreted IL-1 receptor antagonist (IL-1RA) and the membrane-exposed Fas (105).

## Outlook

The recent surge of deciphering neutrophil heterogeneity has started to elucidate the exciting biology of this far less simplistic cell population than previously believed. Especially in the context of diseases such as COVID-19, it has become clear that knowledge about the different neutrophil subpopulations is needed to understand pathology. Clearly, much more work is necessary to comprehensively connect the transcriptionally described subpopulations to cellular functions as well as their role in etiologies and disease outcomes.

## Author Contributions

All authors contributed to the article and approved the submitted version.

## Funding

AA was supported by an intramural grant from the Department of Genomics & Immunoregulation at the LIMES Institute and by an ImmunoSensation² Women in Science grant. The work of JS was supported by the German Research Foundation (DFG) under Germany’s Excellence Strategy (EXC2151 – 390873048), the EU project SYSCID (grant number 733100), the BMBF-funded grant iTREAT (01ZX1902B), ERA CVD (grant number 00160389), and the BMBF-funded excellence project Diet–Body–Brain (DietBB) (grant number 01EA1809A).

## Conflict of Interest

The authors declare that the research was conducted in the absence of any commercial or financial relationships that could be construed as a potential conflict of interest.

## Abbreviations

COVID-19, Coronavirus disease 2019; GMP, granulocyte-monocyte progenitor cell; Neu, neutrophil; ISG, interferon-stimulated genes; NET, neutrophil associated extracellular trap; ROS, reactive oxygen species; MDSC, granulocytic-myeloid derived suppressor cells; LDNs, low-density neutrophils; NE, neutrophil elastase.
